# The Influence of Temper on Health

**Published:** 1863-11

**Authors:** 


**The Influence of Temper on Health.**—Excessive labor, exposure to wet and cold, deprivation of sufficient quantities of necessary and wholesome food, habitual bad lodging, sloth, and intemperance, are all deadly enemies to human life; but they are none of them so bad as violent and ungoverned passions. Men and women have survived all these, and at last reached an extreme old age; but it may be safely doubted whether a single instance can be found of a man of violent and irascible temper, habitually subject to storms of ungovernable passion, who has arrived at a very advanced period of life. It is, therefore, a matter of the highest importance to every one desirous to preserve "a sound mind in a sound body," so that the brittle vessel of life may glide down the stream of time smoothly and securely, instead of being continually tossed about amidst rocks and shoals which endanger its existence, to have a special care, amidst all the vicissitudes and trials of life, to maintain a quiet possession of his own spirit.

